# Formation of amyloid loops in brain tissues is controlled by the flexibility of protofibril chains

**DOI:** 10.1073/pnas.2216234120

**Published:** 2023-05-15

**Authors:** Alyssa Miller, Jiapeng Wei, Sarah Meehan, Christopher M. Dobson, Mark E. Welland, David Klenerman, Michele Vendruscolo, Francesco Simone Ruggeri, Tuomas P. J. Knowles

**Affiliations:** ^a^Yusuf Hamied Department of Chemistry, University of Cambridge, Cambridge CB2 1EW, United Kingdom; ^b^Nanoscience Centre, Department of Engineering, University of Cambridge, Cambridge CB3 0FF, United Kingdom; ^c^Laboratory of Organic Chemistry, Department of Agrotechnology and Food Sciences, Wageningen University & Research, Wageningen 6703 WE, the Netherlands; ^d^Physical Chemistry, Department of Agrotechnology and Food Sciences, Wageningen University & Research, Wageningen 6703 WE, the Netherlands; ^e^Cavendish Laboratory, Department of Physics, University of Cambridge, Cambridge CB3 0HE, United Kingdom

**Keywords:** protein aggregation, amyloid pore, atomic force microscopy

## Abstract

Neurodegenerative diseases, such as Alzheimer’s disease (AD), are associated with protein misfolding and aggregation into amyloid fibrils. Increasing evidence suggests that soluble, low-molecular-weight aggregates play a key role in disease-associated toxicity. Within this population of aggregates, closed-loop pore-like structures have been observed for a variety of amyloid systems, and their presence in brain tissues is associated with high levels of neuropathology. However, their mechanism of formation and relationship with mature fibrils have largely remained challenging to elucidate. Here, we use atomic force microscopy and statistical theory of biopolymers to characterize amyloid ring structures derived from the brains of AD patients. We analyze the bending fluctuations of protofibrils and show that the process of loop formation is governed by the mechanical properties of their chains. We conclude that ex vivo protofibril chains possess greater flexibility than that imparted by hydrogen-bonded networks characteristic of mature amyloid fibrils, such that they are able to form end-to-end connections. These results explain the diversity in the structures formed from protein aggregation and shed light on the links between early forms of flexible ring-forming aggregates and their role in disease.

## Significance

While amyloid pores were first reported nearly two decades ago, their nature and relevance to diseases remain unclear. Here, we characterize loop-like structures in protofibrillar assemblies derived from human Alzheimer’s disease brains. We find that the flexibility of such ex vivo protofibrils enables their two ends to meet to form pore-like structures. The ends of the chains can also dissociate to adopt extended conformations such that they can exist in both open and closed states. These findings suggest that the formation of amyloid loops is a generic process, governed by the fact that intermediate protofibrillar species behave as semiflexible polymers, thus adding to our overall understanding of the formation of disease-relevant protein assembly states.

The study of protein aggregation and amyloid formation has garnered significant attention over the past few decades due to its role in human disease. This includes the aggregation of amyloid-*β* (A*β*) peptide, which is fundamentally implicated in the onset of Alzheimer’s disease (AD) (1, 2). Aggregation broadly involves the conversion of soluble monomers into insoluble amyloid fibrils which possess extended, highly ordered cross-*β* structure (1, 2). Current evidence points toward less organized, low-molecular-weight protofibrillar species as being pathogenic structures (3–6). Such species perturb healthy biological function, from disrupting cellular homeostasis by permeabilizing biological membranes and nonspecific receptor binding to inducing inflammation and cell death (3, 6, 7). This is consistent with the observation that soluble aggregates are associated with cognitive impairment in both mice and human models (8, 9). Their disease relevance has been further highlighted recently with the development of a protofibril-specific monoclonal antibody, lecanemab, which was shown to reduce amyloid burden in brains and slow cognitive decline in slow cognitive decline in a clinical trial (10).

Within this class of low-molecular-weight aggregates, ring-like loop structures have been reported for more than 10 different polypeptide systems, with typical outer diameters in the 6- to 12-nm range (11). This observation has resulted in much attention focusing on their disease relevance and potential mechanisms of action, such as the suggestion that they may behave similarly to bacterial pore-forming toxins (11). However, both the pathways by which they form as well as their relationship with mature fibrils have remained elusive. Much of our understanding comes from in vitro characterization studies performed on recombinantly or synthetically produced proteins (11–13). Thus, it is unclear how these observations correlate with the properties of aggregates present in diseased tissues. Recently, loop structures were reported in soluble fractions from the hippocampus of AD patient brains and characterized using a range of biochemical, biophysical, and cellular assays (5). These structures were associated with toxic effects, from increased levels of inflammation, membrane permeability, and cell death (7). However, unraveling the specific conformation(s) related to diseases is a key challenge.

Here, we employ single-molecule imaging and statistical theory of polymers to characterize the structure and mechanical properties of AD brain-derived and in vitro protofilament and protofibril species in both closed-loop and open conformations, with the aim to understand the forces governing loop formation, and how these protofibrillar species compare to mature amyloid fibrils. To reach this objective, we measure the morphological and mechanical properties of brain-derived (ex vivo) prefibrillar aggregates by atomic force microscopy (AFM) and transmission electron microscopy (TEM). Then, additionally using amyloid assemblies formed by bovine *α*-lactalbumin as a model system, we measure the arc-length distribution of filament and loop structures and show that a theory of semiflexible polymers quantitatively predicts the range over which the formation of closed rings is expected to be favored over open protofibril chains. Finally, we demonstrate that this type of model can be extended to other systems and provides a general framework which links the mechanical properties with the morphology and toxicity of amyloid structures.

## 1. Characterization of Amyloid Loops

**Fig. 1.**
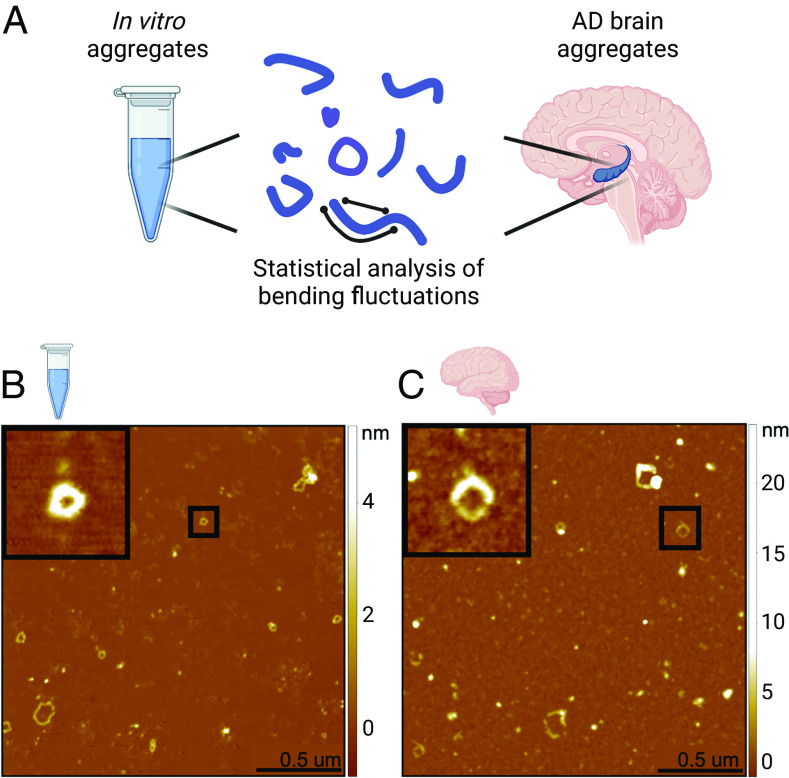
AFM images of in vitro and ex vivo loops. (*A*) Protofilaments and protofibrillar aggregates were extracted and studied by statistical analysis of their bending fluctuations. Samples analyzed were recombinant *α*-lactalbumin chain-like aggregates (in vitro) and soluble aggregates extracted from the hippocampus of AD patient brains (ex vivo). (*B*) Example AFM images are shown for in vitro and (*C*) ex vivo samples. The black squares show a zoom of examples of closed ring structures.

We first induced the formation of elongated aggregates capable of forming rings from recombinant *α*-lactalbumin in vitro (Fig. 1*A*, *SI Appendix*, Fig. S1*A*) Conditions were selected to destabilize the native state of the protein, namely low pH (2.0) and high temperature (50 °C). Initially, nonfibrillar aggregates were observed, followed by the formation of chain-like aggregates after 2 h of incubation (*SI Appendix*, Fig. S2*A*). Amyloid loops and open-chain protofilaments and protofibrils were identified after incubation times ranging from 5 to 14 h (*SI Appendix*, Fig. S2 *B* and *C*). After prolonged incubation at high temperature (72 h, *SI Appendix*, Fig. S2*D*) the filaments and loops were no longer observed, and only aggregates of smaller size were seen, suggesting that the flexible chains possess a low level of structural robustness, an observation in agreement with the mechanical characterization presented below. Decreasing the incubation temperature slowed the kinetics of amyloid fibril formation by *α*-lactalbumin, consistent with the presence of a free-energy barrier resulting from the structural reorganization required associated with the conversion of the protein to the amyloid form. For example, amyloid rings were observed after 1 d of incubation at 37 °C and after 1 mo of incubation at −5 °C. This contains information on the combined nucleation and growth rates, giving an order of magnitude estimate, which is comparable to that measured for the growth of insulin fibrils (14). For the sake of simplicity, protofibrils formed from *α*-lactalbumin will henceforth be referred to as “in vitro” samples.

We then extracted and characterized soluble aggregates from the hippocampus of postmortem Alzheimer’s disease brains. Briefly, brain homogenate was subjected to successive rounds of buffer soaking and centrifugation, and then, the soluble fraction was retrieved and deposited on a mica surface and characterized using AFM (5, 15). We observe heterogeneous structures, including spherical oligomers, protofilaments, and protofibrillar structures which can exist in both a closed-loop and an open conformation, similar to those observed in the in vitro system (Fig. 1*C*, *SI Appendix*, Fig. S1*B*).

## 2. Mechanical Properties of Amyloid Loops

**Fig. 2.**
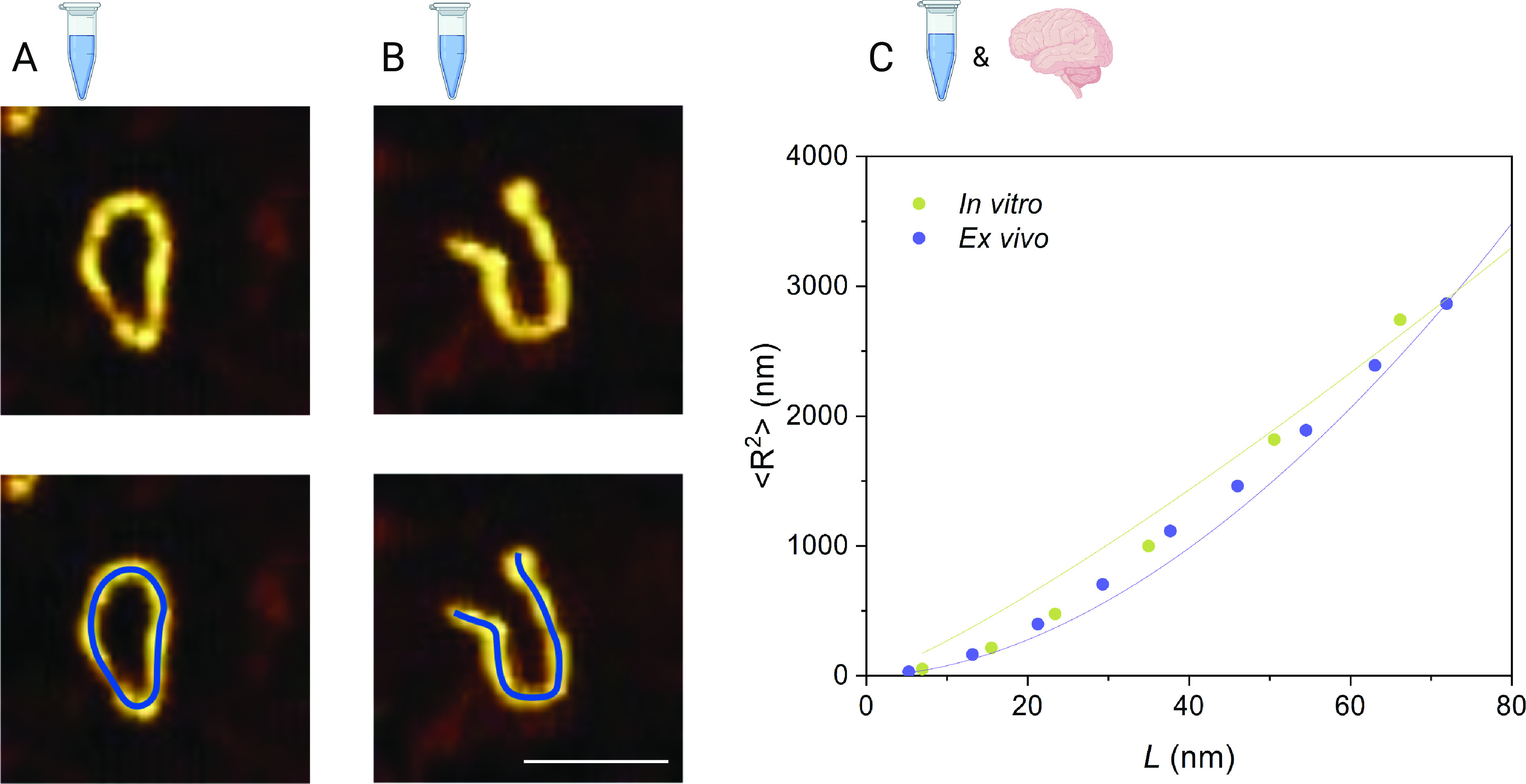
Measuring protofibrils to calculate the persistence length. (*A*) Closed and (*B*) open rings are observed for *α*-lactalbumin. Protofilaments and protofibrils were traced using a semiautomated algorithm by fitting along the aggregate spline (blue line). (Scale bar, 100 nm.) (*C*) Persistence length *l*_*p*_ is calculated based on the plot of ⟨*R*^2^⟩ versus the contour length *L*, as measured from the AFM maps. Green and blue solid lines represent the fit to Eq. **1**. *l*_*p*_ values were found to be 20 nm for in vitro (*n* = 426, 161 images) and 21 nm for ex vivo protofibrils (*n* = 24, 41 images).

Having established the presence of ring-like protofilament and protofibril structures for the in vitro and ex vivo samples, we sought to define their length distribution. Over 1,000 individual structures from over 200 AFM maps were individually traced (Fig. 2) using a semiautomatic algorithm (*Materials and Methods*), and the arc lengths were measured from the digitized contours using fitted splines. Additionally, the height was measured for the same structures by considering the maximal height from the local average background from sections perpendicular to the tangent of the spline following the contour of the filament (*SI Appendix*, Fig. S3). These measurements allow us to understand the mechanical properties of chain-like aggregates, such as the persistence length *l*_*p*_, which can be calculated directly from AFM maps using the mean square end-to-end distance *R* as a function of contour length *L* (Fig. 2*E*):

[1] $$\begin{array}{c} \left\langle R^{2}\right\rangle =2\xi Ll_{p}\left[1-\frac{\xi l_{p}}{L}\left(1-e^{-\frac{L}{pl_{p}}}\right)\right]. \end{array}$$

*ξ* is a surface parameter, which has a value of *ξ* = 2 for a chain that has equilibrated on the surface and 1.5 < *ξ* < 2 for a chain that has been adsorbed without equilibration (16, 17). We first test whether the chains are equilibrated in 2D (i.e., on the surface) by considering in the limit *L* ≫ *l*_*p*_ the scaling exponent *γ* relating contour length *L* to end-to-end distance $\sqrt{{\left\langle R\right\rangle }^{2}}∼L^{\gamma }$. The value of the scaling exponent was found to be 0.72 and 0.75 for ex vivo and in vitro protofibrils, respectively, which is consistent with 2D equilibration (*γ* = 3/4) (*SI Appendix*, Fig. S4). Therefore, the surface value *ξ* = 2 is appropriate here.

From Eq. **1**, we estimated the *l*_*p*_ values for in vitro and ex vivo protofilaments and protofibrils. In the case of the in vitro aggregates, the more uniform nature of the sample allows us to determine a single value of *l*_*p*_ of ∼20 nm. For the ex vivo aggregates, the heterogeneity of human samples means that one value of *l*_*p*_ does not accurately represent the sample. As such, we performed a grouped analysis of the elongated aggregates as a function of their height, which is indicative of their aggregation maturity (18, 19). The groups we selected are 0 to 3 nm, 3 to 6 nm, 6 to 9 nm, and 9 to 12 nm, which corresponds with the height of protofilament chains (*SI Appendix*, Fig. S5). The majority of ring-like structures were found in the 3- to 6-nm height group, with an *l*_*p*_ of ∼21 nm. We used this group as representative of the flexible ex vivo protofibril sample as it corresponds well with the height observed for in vitro protofibrils, facilitating comparison. We next calculated the bending rigidity (*C*_*B*_) from the values of *l*_*p*_ from *C*_*B*_ = *K*_*B*_*Tl*_*p*_, which were found to be 8.5 × 10^−29^ N/m for ex vivo protofibrils and 8.1 × 10^−29^ N/m for in vitro protofibrils. These values are significantly lower than those reported for mature amyloid fibrils, (20), indicating a lack of intermolecular hydrogen-bonding and extended cross-*β* structure (19, 21, 22).

## 3. Modeling the Probability of Loop Closure

**Fig. 3.**
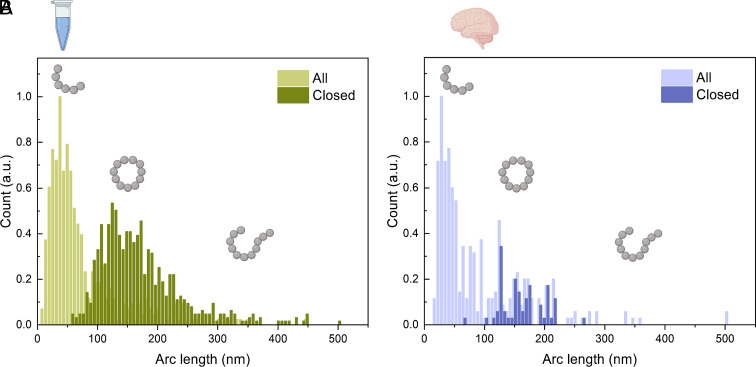
Arc-length distributions of both open and closed conformations. Distributions of both open and closed conformations were measured for (*A*) in vitro (*n* = 1022, 161 images) and (*B*) ex vivo (*n* = 324, 41 images) protofibrils.

Having characterized the mechanical properties of protofibrils, we next sought to understand the likelihood of finding a protofibril in a closed or open conformation. We measured the incidence of closure as a function of the arc length, *s*, where *s* follows the contour of the curve from 0 to the full contour length *L*. The data in Fig. 3 show that for small values of the arc length, all protofibrils are found as open chains; with increasing arc length, the probability of finding loops increases, and finally, for very long chains, it decreases again. In order to interpret these measurements in a quantitative way, we consider a simple model based on statistical theory of biopolymers. At any given time, a fibril composed of *j* monomers can exist either as an open-chain configuration with two free ends or in a closed-loop state where the ends are joined. These two forms are interconverted with rates that in general depend on the length of the chains: $\frac{d}{\mathrm{dt}}R\left(j\right)=-R_{j}k_{R\to F}\left(j\right)+F_{j}k_{F\to R}\left(j\right)$ where *F*_*j*_ and *R*_*j*_ are the concentrations of the open (*F*, free) and closed (*R*, ring) forms, and *k*_*F* → *R*_(*j*) and *k*_*R* → *F*_(*j*) are the first-order rates for ring closure and opening.

We first consider the ring closure rate: *k*_*F* → *R*_(*j*) = $k_{F\to R}^{0}$ ⋅ *g*(*j*), whereby the *j* dependence is in the circularization probability *g*(*j*), and the constant factor $k_{F\to R}^{0}$ contains information about the diffusion coefficient of the ends of the polymer as well as about the reaction cross-sections. The circularization probability for semiflexible chains has been explored theoretically by Stockmayer and Yamakawa (23) and by Liverpool and Edwards (24) and is very generally given by the sum of the Boltzmann weights corresponding to different configurations of the bond vectors $\left\{{\vec{u}}_{1}\cdots {\vec{u}}_{j}\right\}$, which satisfy the loop closure condition $\sum_{j=1}^{N}{\vec{u}}_{i}=0$:

[2] $$\begin{array}{c} g\left(j\right)=\frac{\int d{\vec{u}}_{1}\cdots \int d{\vec{u}}_{j}\;\delta \left(\sum_{j=1}^{N}{\vec{u}}_{i}\right)e^{-\beta H(\left\{{\vec{u}}_{1}\cdots {\vec{u}}_{j}\right\})}}{\int d{\vec{u}}_{1}\cdots \int d{\vec{u}}_{j}\;e^{-\beta H(\left\{{\vec{u}}_{1}\cdots {\vec{u}}_{j}\right\})}}, \end{array}$$

with the semiflexible Hamiltonian: $H(\left\{{\vec{u}}_{1}\cdots {\vec{u}}_{j}\right\})$ = $\frac{C_{B}}{b_{0}}\sum_{i=1}^{j}$$\left(1-{\vec{u}}_{i}\cdot {\vec{u}}_{i+1}\right)$, where *C*_*B*_ = *K*_*B*_*Tl*_*p*_ is the bending rigidity, and *b*_0_ = *s*/*j* = *L*/*N* is the bond length, corresponding to the distance between *β*-strands. *β* = 1/*K*_*B*_*T* is the reciprocal of the thermodynamic temperature of a system. We take here the result based on perturbation theory derived by ref. 23, which enables an approximate value of the circularization probability to be derived for semiflexible chains in the continuum limit; the result can be expressed as a simple function of a scaled dimensionless arc length, $q=\frac{s}{2\beta C_{B}}=\frac{s}{2l_{p}}$ denotes the renormalized dimensionless arc length:

[3] $$\begin{array}{c} g\left(q\right)\propto \left\{\begin{array}{cc} \frac{C_{0}}{q}e^{-A/q}\left(1-C_{1}q\right) & q\leq \sigma \\ \frac{1}{q^{3/2}}\left(1-\frac{5}{8}\frac{1}{q}-\frac{79}{640}\frac{1}{q^{2}}\right) & q>\sigma , \end{array}\right. \end{array}$$

where the approximate numeric values of the dimensionless constants are *σ* = 0.96, *C*_0_ = 1.51 ⋅ 10^3^, *C*_1_ = 0.81, and *A* = 7.026, as determined in ref. 23.

We furthermore include the possibility for the two ends of the chain to break apart again, and this process can be modeled as a thermally activated barrier crossing, where in the general case, the barrier height depends on the strain induced by the ring closure condition: *ΔG*_opening_ = *ΔG*_0_ − *G*_*el*_(*j*), where *ΔG*_0_ is the free-energy barrier in the absence of strain and $G_{\mathrm{el}}\left(j\right)=\frac{l_{p}}{\beta b_{0}}\left(1-{\vec{u}}_{i}\cdot {\vec{u}}_{i+1}\right)$ is the elastic energy per bond of *j* monomers protofibril with length *b*_0_ due to the strain of the imposed loop configuration and is given by the difference in the orientation of the unit bond vectors ${\vec{u}}_{i}$ and ${\vec{u}}_{i+1}$ (25). We assume that the system has the circular shape ${\vec{u}}_{i}\cdot {\vec{u}}_{i+1}$ = $\cos(\frac{2\pi }{j})$ and approximate *G*_*el*_(*j*) ≈ 2*π*^2^*C*_*B*_/*j*^2^*b*_0_ for *j* ≫ 1. Since there are *j* − 1 bonds that can break in a closed loop of *j* monomers, the opening rate can be written as *k*_*R* → *F*_(*j*) ≈ $k_{R\to F}^{0}$ ⋅ *j* ⋅ *e*^2*π*^2^*βC*_*B*_/*j*^2^*b*_0_^, where $k_{R\to F}^{0}$ is a constant which contains information on the absolute value of *ΔG*. We further substitute *s* = *b*_0_*j* for data analysis.

**Fig. 4.**
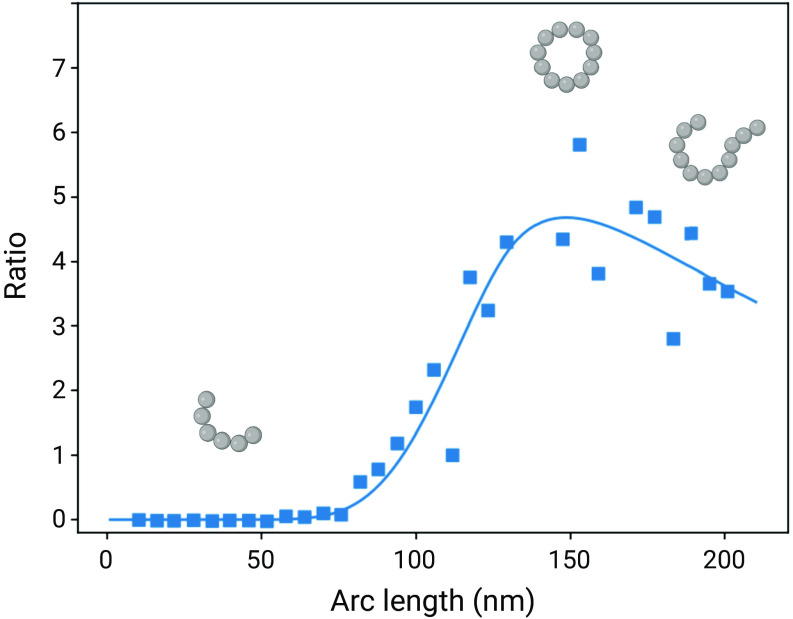
The probability of loop closure as a function of the arc length. The probability of loop closure was plotted as a function of protofibril length, based on theory of semiflexible polymers. The y-axis reports the ratio of the opening and closing rates. Blue data points are experimental ratios of open:closed for the in vitro protofibrils. The blue line is a fit from Eq. **4**. As the arc length increases, so does the likelihood of finding a protofibril in a closed-loop conformation. Above a certain length, the likelihood decreases again as the probability of two open-chain ends finding each other decreases.

If the system is in a steady state with respect to the conversion between open ended filaments and loops, the probability *P*(*s*) of finding a given structure in the closed configuration as a function of the arc length *s* is given by *P*(*s*) = *r*(*s*)/(*r*(*s*)+1), where *r*(*s*) is the ratio between the closing and opening rates (Fig. 4):

[4] $$\begin{array}{cc} r(s) & =\frac{b_{0}}{s}\cdot \frac{k_{F\to R}^{0}}{k_{R\to F}^{0}}\;e^{-\frac{2\pi^{2}\beta C_{B}b_{0}}{s^{2}}}\\ & \;\cdot \left\{\begin{array}{cc} C_{0}\cdot \frac{2\beta C_{B}}{s}\cdot e^{-\frac{2\beta C_{B}A}{s}}\left(1-C_{1}\frac{s}{2\beta C_{B}}\right) & s\leq 2\beta C_{B}\sigma \\ {\left(\frac{2\beta C_{B}}{s}\right)}^{3/2}\left(1-\frac{5}{4}\frac{\beta C_{B}}{s}-\frac{79}{160}\frac{\beta^{2}C_{B}^{2}}{s^{2}}\right) & s>2\beta C_{B}\sigma . \end{array}\right. \end{array}$$

We assume that the protofibril can break at any location between two individual *β* strands. We approximate *b*_0_ = 0.48 nm and temperature *T* = 323K. Using Eq. **4**, we performed a two parameter fit to the measurements in Fig. 3, were the unit-less prefactor *Ψ* = $k_{F\to R}^{0}$/$k_{R\to F}^{0}$ and the bending rigidity *C*_*B*_ are left free yields the value *Ψ* = 5278 and *C*_*B*_ = 3.05 ⋅ 10^−28^ Nm^2^, which is in good agreement with the value obtained by statistical analysis of AFM images. This value of the bending rigidity controlling the loop formation is also of a similar order of magnitude as that found for the open chains of *α*-lactalbumin of *C*_*B*_ = 1.4 ⋅ 10^−28^ Nm^2^ from an independent analysis of shape fluctuations (14), showing that the intrinsic mechanical properties of the filaments determine the distribution of ring structures.

The present measurements also enable us to investigate the energetics of the ring closure. There is an enthalpic energy cost of chain bending to bring the two ends together, governed by the flexibility and diameter, *d*, of the loop, which is represented by *ΔH* = 2*C*_*B*_*π*/*d* > 0. Therefore, the energy gain from the ligation of the ends must exceed this value for this state to be significantly populated. We observe loops down to a diameter of approximately *d* = 19 nm, and therefore, the interaction between the filament ends must contribute at least 11 kcal/mol, an energy which is equivalent to that gained from the formation of 2 to 3 hydrogen bonds. The mechanical strain within the smallest loops can be estimated from geometric considerations: *ϵ* = *Δl*/*l*_0_ = *h*/*d* ≈ 5.2%, where *h* is the height of the filaments, and *d* the diameter of the ring. This value is smaller than the maximal strain which the cross-*β* core structure in insulin fibrils was shown to be able to accommodate without fracturing (18%) (26).

**Fig. 5.**
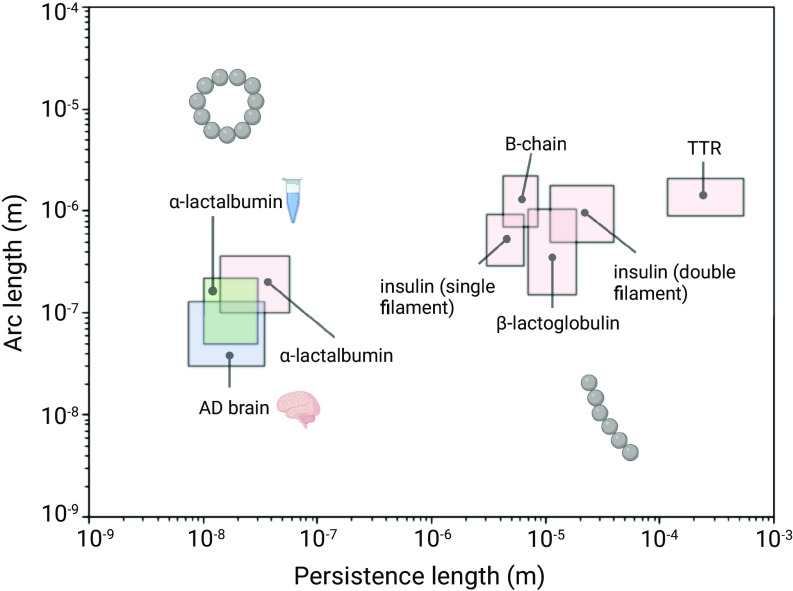
Persistence length plotted as a function of arc length for a variety of amyloid protofibril and fibril assemblies. The persistence length and arc length are plotted for a variety of amyloid systems. From the present work are ex vivo aggregates and in vitro (*α*-lactalbumin) aggregates, indicated by the brain and test tube, respectively. Literature values are plotted for *α*-lactalbumin, insulin (single filament), B-chain, *β*-lactoglobulin, insulin (double filament), and TTR from ref. 14. Where the ratio between persistence length and arc length is below one, loop structures may form.

Finally, we can contextualize our findings by considering the measurements of the rigidities of other amyloid fibrils. For a variety of fibril systems from the literature (14), we plotted the persistence length vs the arc length (Fig. 5). We can intuitively understand that structures which have a ratio of persistence length to arc length less than or equal to one may exist in the loop configuration, whereas structures with a ratio significantly above one exist only as linear fibers. The values of persistence length reported here are below the values typically found for mature amyloid fibrils, suggesting that ex vivo and in vitro protofibrils have a lower level of structural organization than that which is generally found in amyloid fibrils.

## 4. Discussion

We have described the mechanical properties of brain-derived protofibrils and the forces that govern their loop formation in order to increase our understanding of the nature of loop structures in neurodegenerative diseases. We have demonstrated that human AD brain-derived protofibrillar structures possess significantly lower structural rigidity compared to mature amyloid fibrils. The mechanical properties of mature amyloids are imparted by their extended intermolecular hydrogen-bonding networks (20, 27–30). The values of bending rigidity found here are orders of magnitude lower than those typically reported for amyloid fibrils. This is consistent with lower order and density of cooperative *β*-sheet bonding networks, as has been reported in the literature for in vitro protofibrils (21, 31). This structural flexibility is what allows protofibrils to exist as loop-like structures, via the formation and breakage of end-to-end connections. Then, the likelihood of finding a protofibril in a closed loop can be simply understood as the probability of the two ends finding each other.

Previously reported in vitro loop structures were more homogeneous in size, leading researchers on the search for a uniform, rigid class of structures, such as a pore-forming hexamer (12, 13, 32). This is inconsistent with the data presented here on brain-derived aggregates, which are heterogeneous both in morphology and whether they are found in a loop or stretched-out conformation. This adds evidence to the idea that the search for a single toxic species in AD may be a fruitless one and that rather, a heterogeneous, dynamic population of low-molecular-weight structures is involved in disease pathogenesis (6).

While we have not investigated the mechanism of action of soluble protofibrils, this has been investigated in previous work, making it possible to draw links between the mechanical properties established here and their biological function (5). It is clear that these structures capable of forming loops are distinct from mature amyloid fibrils found in plaques of AD brains, not only in size and solubility but also in internal structural properties. These differences in mechanical properties not only explain the ability certain amyloid states possess to form loops but may also explain the discrepancies in mechanisms of actions between mature and earlier, protofibrillar structures. These soluble, low-molecular-weight species were associated with increased levels of inflammation, membrane permeability, and cell death, suggesting that these are disease-relevant structures (5). This toxicity may be imparted by their low level of structural robustness, such that they are more capable of forming nonspecific, aberrant interactions with cell membranes and receptors.

## Materials and Methods

### Preparation of Endogenous Aggregates from Human Brains.

Endogenous aggregates were extracted from Alzheimer’s disease as previously described (5). Briefly, these experiments employ aggregates derived from fresh frozen brains from three Alzheimer’s disease patients diagnosed as being at Braak stage III. Soluble aggregates were obtained by following a previously established protocol with a few adaptations (4). Human brain tissue was chopped into 300-mg pieces using a razor blade and incubated with gentle agitation in 1.5 mL of artificial cerebrospinal fluid (aCSF) buffer (124 mM NaCl, 2.8 mM KCl, 1.25 mM *NaH*_2_*PO*_4_, and 26 mM *NaHCO*_3_, pH 7.4, supplemented with 5 mM EDTA, 1 mM EGTA, 5 μg/mL leupeptin, 5 μg/mL aprotinin, 2 g/mL pepstatin, 20 μg/mL Pefabloc, and 5 mM NaF) at 4 °C for 30 min. Samples were centrifuged at 2,000 g at 4 °C for 10 min, and the upper 90% of the supernatant was collected and centrifuged at 14,000 g for 110 min at 4 °C. The upper 90% of the supernatant was extracted and dialyzed using Slide-A-Lyzer cassettes (Thermo Scientific, Cat. 66330) with a 2-kDa molecular weight cutoff, against 100-fold excess of fresh aCSF buffer with gentle agitation at 4 °C. Buffer was changed three times over the course of 72-h dialysis. The prep was carried out under sterile conditions, using autoclaved LoBind Eppendorf tubes and presterilized pipette tips to reduce endotoxin contamination. Samples were aliquoted into small volumes, snap-frozen, and stored in a −80 °C freezer and thawed only once prior to experimentation.

### Formation of Amyloid Fibrils by Bovine α-Lactalbumin.

*α*-Lactalbumin from bovine milk (Type III, calcium depleted, Sigma-Aldrich) was dissolved at 5 mg/ml in *H*_2_*O* adjusted to pH 2.0 with HCl, with 20 mM dithiothreitol (DTT), and incubated at 50 °C. After 3 to 6 h incubation at 50 °C, amyloid fibrils, both in the form of open chains and loops were identified by TEM and further confirmed by a positive interaction with the dyes Thioflavin-T and Congo Red. Amyloid fibril formation preceded more slowly at lower incubation temperatures: Amyloid loops were visible by TEM after 1-d incubation at 37 °C, after 1 mo at 5 °C.

### Transmission Electron Microscopy (TEM).

Transmission electron microscopy (TEM) nickel electron microscopy grids with carbon-coated formvar support films were prepared for microscopy by the addition of 2 l of protein sample at a concentration of 1 mg/ml. The grids were then washed with 3 × 10 μl *H*_2_*O* and negatively stained with 10 μl of uranyl acetate (2% (w/v), Agar Scientific). Samples were viewed under 20125-K magnifications at 120-kV acceleration voltages using a Philips CM100 transmission electron microscope.

### Atomic Force Microscopy (AFM).

*α*-lactalbumin protein solution (0.05 to 1.0 mg/ml) was deposited onto freshly cleaved mica substrates (Agar Scientific Ltd., Stansted, Essex) and air-dried. Topographic data were then acquired with a Dimension 3100 SPM (Veeco Instruments Inc., Woodbury, NY, USA) in conjunction with a Nanoscope IV control system operating in tapping mode. Ultrasharp MikroMasch silicon cantilevers (NSC12/Si3N4/50) were used at resonance frequencies between 150 kHz and 225 kHz.

Endogenous protein imaging was performed in a similar way. Protein solutions were diluted 10x in PBS buffer, and 10 μl was deposited onto freshly cleaved mica. The samples were incubated for 10 min, followed by rinsing with 1 mL milliQ water. The samples were then dried using a gentle flow of nitrogen gas. AFM maps of 3-D morphology were acquired in regime of constant phase change, with 2 to 4 nm/pixel resolution using a NX10 (Park Systems, South Korea) operating in noncontact mode. This setup was equipped with a silicon tip with a nominal radius of < 10 nm and a spring constant of 5 N/m (PPP-NCHR). Scanning Probe Image Processor (SPIP) (version 6.7.3, Image Metrology, Denmark) software was used for image flattening and single aggregate statistical analysis. The average level of noise for each image was measured using SPIP software and was smaller than 0.1 nm. All the measurements were performed at room temperature.

### Statistical Analysis.

The arc length and shape of protofibrils were measured using a semiautomated algorithm that extracts the highest points of the protofibril axis and fits a 2-dimensional parametric spline to these points. The *l*_*p*_ values were then extracted from statistical analysis of these shape fluctuations, as described in the main text, using DNA Trace software (33). The height of protofibrils was measured by considering the average maximal height from the average height of the background and was found to be 2.8 ± 2.3 nm for endogenous aggregates and 1.9 ± 0.4 for synthetic aggregates.

## Supplementary Material

Appendix 01 (PDF)

## Data Availability

All study data are included in the article and/or supporting information.
