# Supplementary material for: Formation of amyloid loops in brain tissues is controlled by the flexibility of protofibril chains
Source: Proc Natl Acad Sci U S A. 2023 May 15;120(21):e2216234120. doi: 10.1073/pnas.2216234120 (PMC10214208; doi:10.1073/pnas.2216234120)
Supplement: Supplementary file 1 — Appendix 01 (PDF) [file pnas.2216234120.sapp.pdf]

PNAS

Supporting Information for

Formation of amyloid loops in brain tissues is controlled by the flexibility of protofibril chains.

Alyssa M. Miller[1], Jiapeng Wei[1], Sarah Meehan[1], Christopher M. Dobson[1], Mark E. Welland[2], David Klenerman[1], Michele Vendruscolo[1], Francesco S. Ruggeri[1,3,4], and Tuomas P. J. Knowles[1,5]

[1]Yusuf Hamied Department of Chemistry, University of Cambridge, Cambridge, CB2 1EW, UK
[2]Nanoscience Centre, University of Cambridge, Cambridge, CB3 0FF, UK
[3]Laboratory of Organic Chemistry, Stippeneng, Wageningen University, Wageningen, 6703 WE, Netherlands
[4] Laboratory of Physical Chemistry, Stippeneng, Wageningen University, Wageningen, 6703 WE, Netherlands
[5]Cavendish Labratory, Department of Physics, University of Cambridge, Cambridge, CB3 0HE, UK

Michele Vendruscolo, Francesco S. Ruggeri, Tuomas P.J. Knowles
Email: mv254@cam.ac.uk, simone.ruggeri@wur.nl, tpjk2@cam.ac.uk

This PDF file includes:

Figures S1 to S5

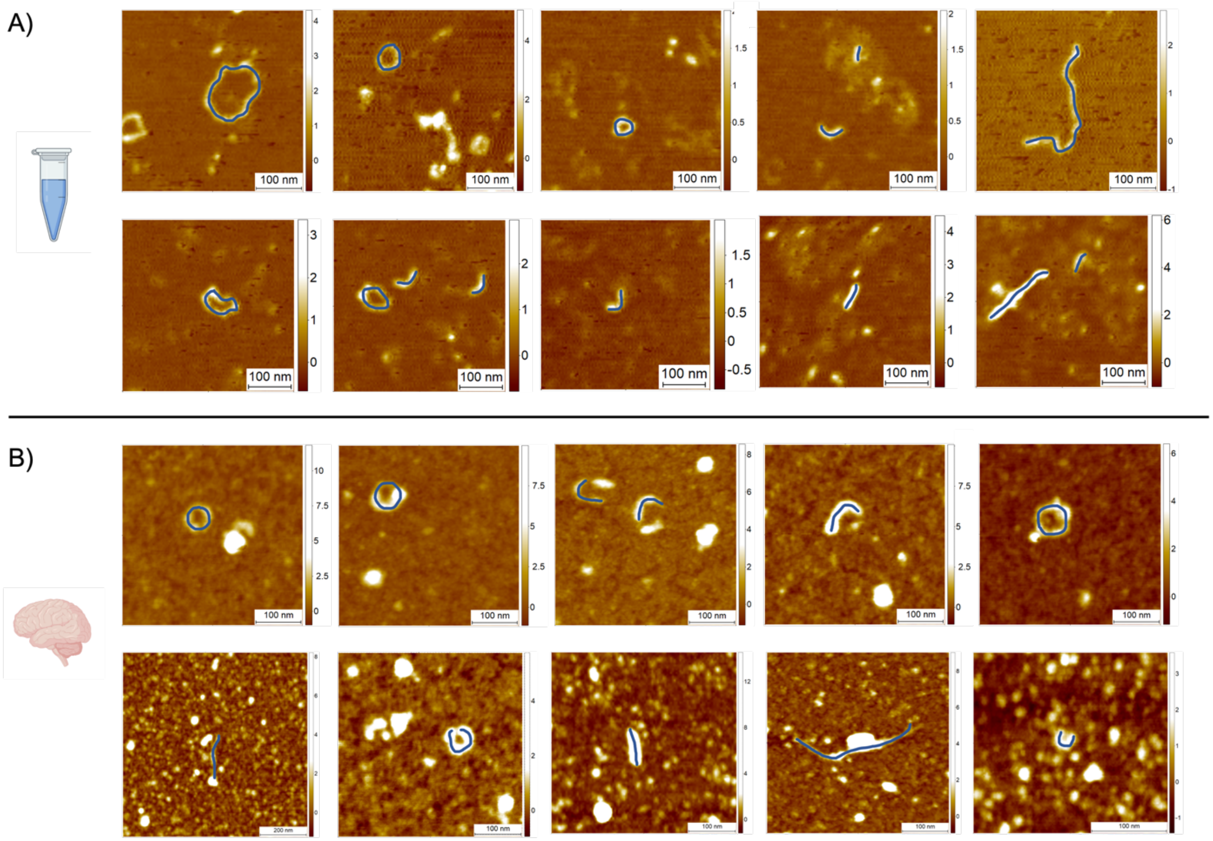

Fig. S1. A gallery of examples of open and closed loops of varying length for *in vitro* (A) and *ex vivo* (B) protofibrils. Loops were traced (blue lines) as a visual aid to demonstrate which particles were included in the analysis.

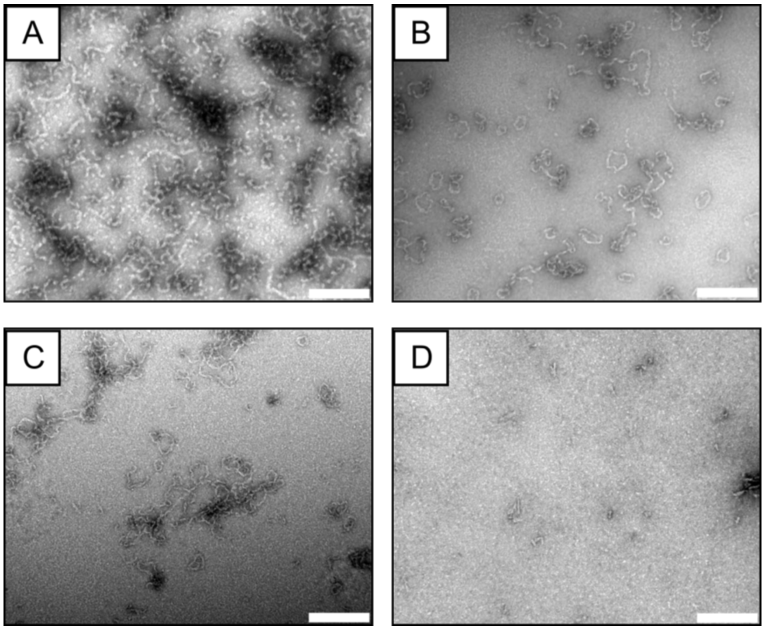

Fig. S2. TEM imaging of recombinant α-lactalbumin aggregates}. (A-D) α-lactalbumin was imaged via TEM in destabilising conditions after 2 (A), 5 (B), 14 (C), and 72 (D) hours incubation time. Protofibrillar structures are initially observed (A), followed by the observation of closed ring structures (B & C). After prolonged incubation (D), the elongated structures are no longer observed.

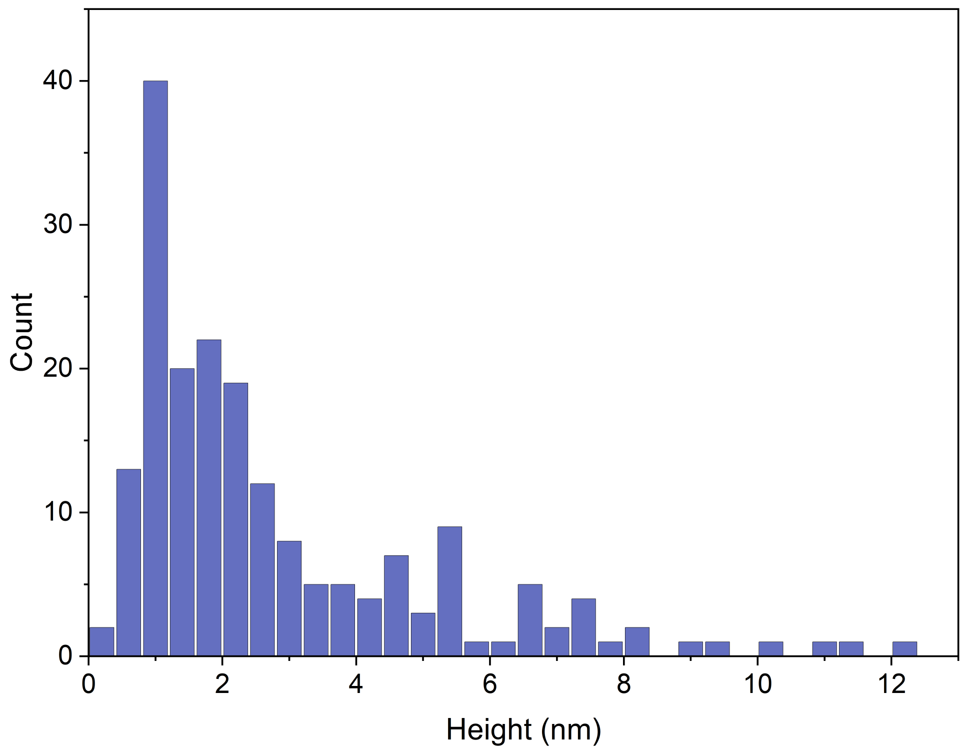

Fig. S3. The cross-sectional height distribution for *ex vivo* aggregates was measured using AFM. The mean height was 2.8 ± 2.3 nm (n=198).

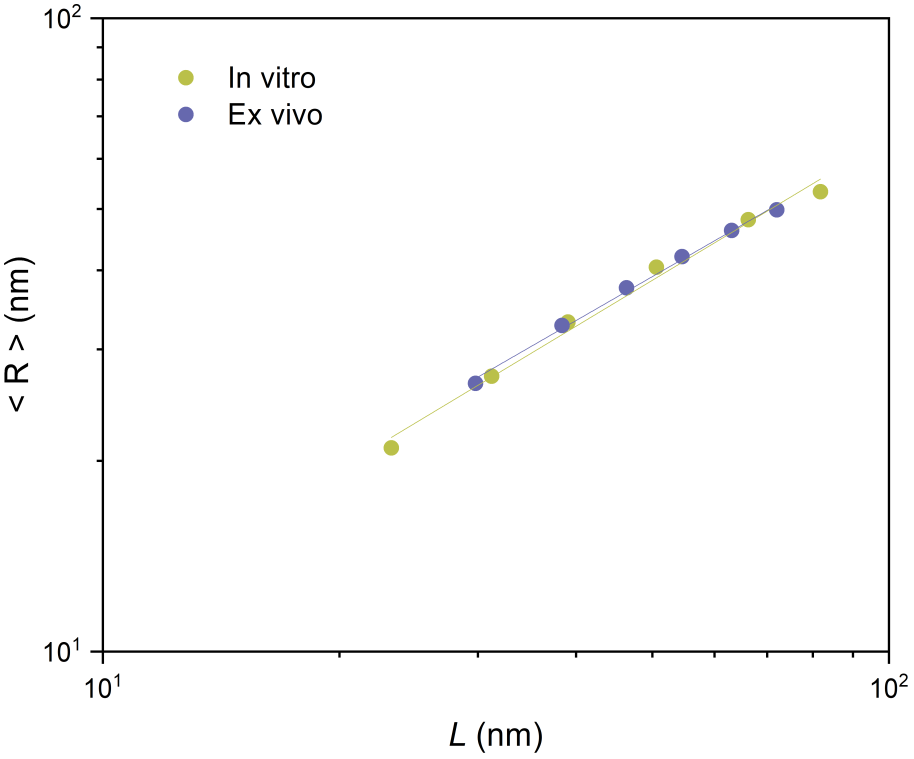

Fig. S4. A double logarithmic plot of the end-to-end distance, R, vs the contour length, L. The scaling exponent, λ, provides information on the dimension of the system, with λ = 1, 0.75, 0.588 for D = 1, 2, and 3 respectively. λ = 0.72 for *ex vivo*, and λ = 0.75 for *in vitro* protofibrils.

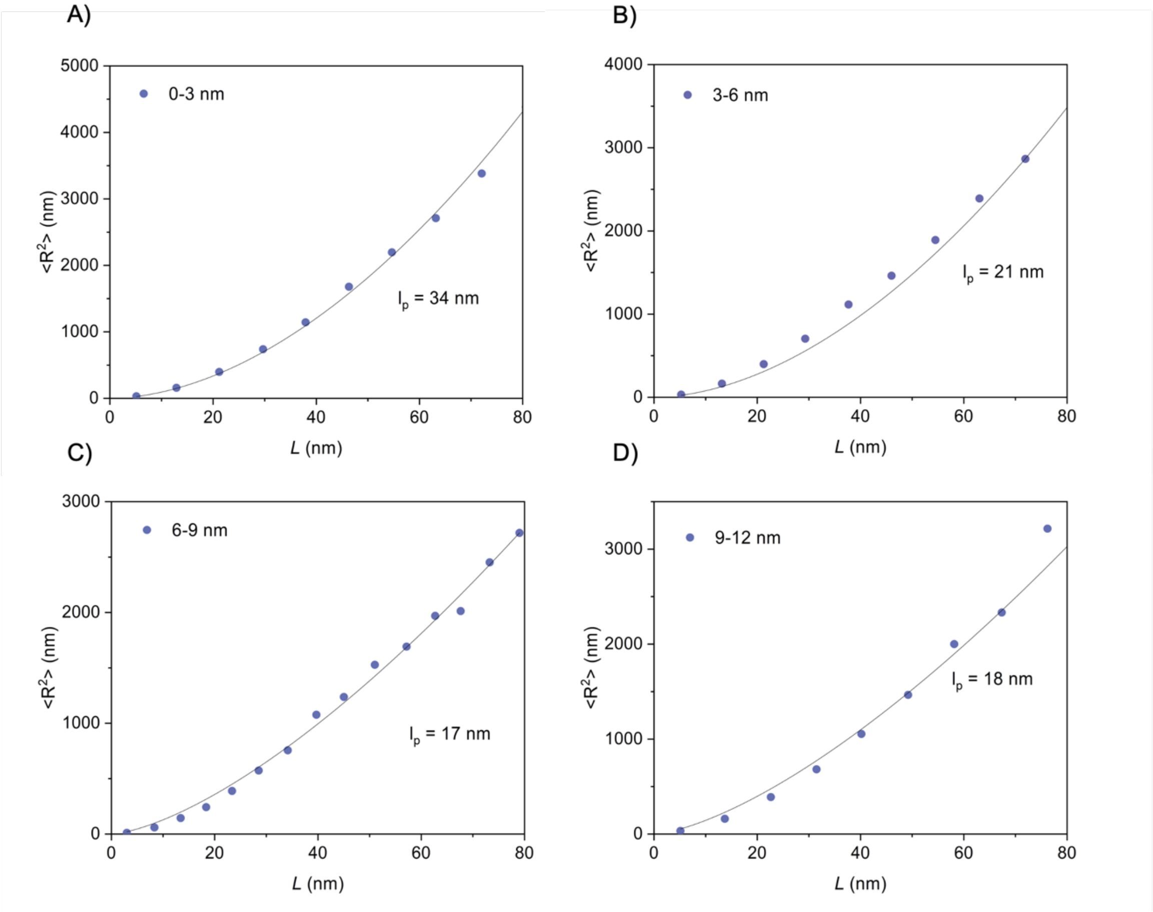

Fig. S5. Plots of contour length, *L* vs mean squared end-to-end distance, $\langle R^2 \rangle$ for *ex vivo* protofibrils. Due to the heterogeneous nature of brain samples, it is likely that more than one protein species exists. Analysis was therefore performed in groups corresponding the height of (A) 1 (n=137), (B) 2 (n=24), (C) 3 (n=28), and (D) 4 (n=9) individual protofilaments.
